# Modulation of TRAIL resistance in colon carcinoma cells: Different contributions of DR4 and DR5

**DOI:** 10.1186/1471-2407-11-39

**Published:** 2011-01-27

**Authors:** Caroline MM van Geelen, Bodvael Pennarun, Phuong TK Le, Elisabeth GE de Vries, Steven de Jong

**Affiliations:** 1 Departments of Medical Oncology and Gastroenterology, University Medical Center Groningen, University of Groningen, Groningen, the Netherlands; 2 Department of Medical Oncology, University Medical Center Groningen, University of Groningen, Groningen, the Netherlands

## Abstract

**Background:**

rhTRAIL is a therapeutic agent, derived from the TRAIL cytokine, which induces apoptosis in cancer cells by activating the membrane death receptors 4 and 5 (DR4 and DR5). Here, we investigated each receptor's contribution to rhTRAIL sensitivity and rhTRAIL resistance. We assessed whether agonistic DR4 or DR5 antibodies could be used to circumvent rhTRAIL resistance, alone or in combination with various chemotherapies.

**Methods:**

Our study was performed in an isogenic model comprised of the SW948 human colon carcinoma cell line and its rhTRAIL resistant sub-line SW948-TR. Effects of rhTRAIL and agonistic DR4/DR5 antibodies on cell viability were measured using MTT assays and identification of morphological changes characteristic of apoptosis, after acridine orange staining. Sensitivity to the different death receptor ligands was stimulated using pretreatment with the cytokine IFN-gamma and the proteasome inhibitor MG-132. To investigate the mechanisms underlying the changes in rhTRAIL sensitivity, alterations in expression levels of targets of interest were measured by Western blot analysis. Co-immunoprecipitation was used to determine the composition of the death-inducing signalling complex at the cell membrane.

**Results:**

SW948 cells were sensitive to all three of the DR-targeting agents tested, although the agonistic DR5 antibody induced only weak caspase 8 cleavage and limited apoptosis. Surprisingly, agonistic DR4 and DR5 antibodies induced equivalent DISC formation and caspase 8 cleavage at the level of their individual receptors, suggesting impairment of further caspase 8 processing upon DR5 stimulation. SW948-TR cells were cross-resistant to all DR-targeting agents as a result of decreased caspase 8 expression levels. Caspase 8 protein expression was restored by MG-132 and IFN-gamma pretreatment, which also re-established sensitivity to rhTRAIL and agonistic DR4 antibody in SW948-TR. Surprisingly, MG-132 but not IFN-gamma could also increase DR5-mediated apoptosis in SW948-TR.

**Conclusions:**

These results highlight a critical difference between DR4- and DR5-mediated apoptotic signaling modulation, with possible implications for future combinatorial regimens.

## Background

Tumor necrosis factor related apoptosis inducing ligand is a member of the tumor necrosis factor (TNF) superfamily. Recombinant human TRAIL (rhTRAIL) is currently drawing attention in the field of cancer therapy because of its specific action in inducing apoptosis in tumor cells. Five TRAIL-receptors have been identified to date. The death receptors DR4 and DR5 transduce the apoptotic signal, while three decoy receptors - decoy receptor (DcR1), decoy receptor 2 (DcR2) and osteoprotegerin (OPG) - block the signal and thereby inhibit TRAIL-mediated apoptosis [1,2]. Administration of rhTRAIL in tumor-bearing animals has been shown to induce significant tumor regression without systemic toxicity [3,4]. Furthermore, rhTRAIL in combination with chemotherapy or radiotherapy greatly enhances anti-tumor efficacy, both *in vitro *and *in vivo *[5-8].

The TRAIL apoptotic pathway can also be stimulated by death receptor (DR)-specific agonistic antibodies. These anti-DR4 and anti-DR5 monoclonal antibodies, either used alone or in combination with chemotherapy (or irradiation), induce apoptosis in tumor cells *in vitro *and *in vivo *[9-12]. Thus, both rhTRAIL and agonistic antibodies exhibit interesting preclinical anti-tumor properties. A phase I clinical study on rhTRAIL has been initiated [13]. Several phase I-II clinical studies on agonistic DR4 antibodies, as well as a phase I study on agonistic DR5 antibodies, have also been performed [2,14,15]. However, because rhTRAIL and DR-agonistic antibodies differently stimulate the apoptotic signaling cascade, drug-specific effects in the treatment of cancer patients are expected [16-18]. rhTRAIL, which can bind to DR4 and DR5 but also to the decoy receptors, triggers cross-linking of these receptors into homo- and/or heterotrimers [19,20]. In contrast, agonistic DR4 or DR5 antibodies have been suggested to trigger the formation of multimeric complexes consisting of only one specific receptor, which consequently enables them to bypass the decoy receptors [21,22].

Not all tumor cells are sensitive to rhTRAIL, since intrinsic or acquired resistance to this ligand can occur. Very little is known about the specific properties of different DR agonists when it comes to the downstream activation signaling pathways (e.g. NFκB) and resistance to rhTRAIL. However, rhTRAIL and agonistic anti-DR5 antibodies are known to exhibit different abilities to induce the conformational changes in DR5 which are required to facilitate FADD recruitment [23].

The cytokine IFN-γ, and also proteasome inhibitors, are both known to modulate components of the apoptotic signaling pathway involved in TRAIL resistance [24-26]. Combinations of these drugs with TRAIL and/or agonistic death receptor antibodies can enhance TRAIL-induced apoptosis and overcome TRAIL resistance in tumor cells [27-32]. However, potential receptor specific effects on the development of resistance to rhTRAIL have not been investigated. This is of interest, as it has not yet been established which of the agents of interest - DR4 antibodies, DR5 antibodies or rhTRAIL - exhibit superior anti-tumor activity in the clinic. Moreover, it is still unknown which biomarkers should be used to select patients for therapies specifically targeting DR4 and DR5.

In the present study we used agonistic monoclonal antibodies to individually evaluate the roles of DR4 and DR5 in rhTRAIL sensitivity and TRAIL resistance. We compared apoptosis induced by rhTRAIL with apoptosis induced by agonistic DR4- and DR5- antibodies, taking as our model an rhTRAIL-sensitive human colon cancer cell line and its rhTRAIL-resistant sub-line [33]. Furthermore, we analyzed whether the effects of IFN-γ and a proteasome inhibitor in modulating apoptosis differed depending on how apoptosis was induced; that is, we compared the modulatory effects of these compounds after using agonistic DR4 and DR5 antibodies, or rhTRAIL, to initiate apoptosis.

## Methods

### Reagents

We used RPMI 1640 medium obtained from Life Technologies (Breda, the Netherlands) and fetal calf serum (FCS) from Bodinco BV (Alkmaar, the Netherlands). 3-(4,5-dimethyl-thiazol-2-yl) 2,5-diphenyltetrazolium bromide (MTT)-solution and CHX were purchased from Sigma-Aldrich Chemie BV (Zwijndrecht, the Netherlands). rhTRAIL was produced non-commercially in cooperation with IQ-Corporation (Groningen, the Netherlands) following a protocol described earlier [34]. To stimulate DR4, an agonistic anti-DR4 antibody (HGS-ETR1) was used. The agonistic anti-DR5 antibodies HGS-ETR2 or TR2J were used to stimulate DR5 with similar results. HGS-ETR1, HGS-ETR2 and TR2J were a kind gift from Human Genome Sciences (HGS, Rockville, MD, USA). The inhibiting anti-DR4 (HS 101) and anti-DR5 (HS 201) antibodies were purchased from Alexis (10 P's BVBA, Breda, the Netherlands). The proteasome inhibitor MG-132 was obtained from Calbiochem (Breda, the Netherlands), and IFN-γ was purchased from Roche Diagnostics (Mannheim, Germany). The TRAIL receptor agonistic antibodies used for flow cytometry were obtained from Immunex Corporation (Seattle, WA, USA).

### Cell lines

The rhTRAIL-sensitive colon carcinoma SW948 cell line [35] was cultured as described previously [36]. The rhTRAIL-resistant SW948-TR cell lines was generated and cultured as described recently [33].

### SDS-polyacrylamide gel electrophoresis and Western blotting

Protein lysate preparation and Western blot analysis were performed as described previously [36].

### Co-Immunoprecipitation of TRAIL-, DR4- or DR5-DISC

DISC immunoprecipitation after TRAIL-receptor ligation was performed according to Bodmer *et al*. [37] with some modifications. Briefly, 50.10^6 ^cells per condition were grown, harvested and collected by centrifugation. The cell pellet was resuspended in 1 ml pre-warmed medium, and the tube was placed in a 37°C incubator. Recombinant human soluble flag-tagged TRAIL and the anti-Flag monoclonal antibody M2 were premixed for 15-30 min on ice. Cells were stimulated in a final volume of 1 ml with 500 ng/ml Flag-tagged TRAIL and 1.5 μg/ml M2. In unstimulated cells, the Flag-tagged TRAIL and M2 premix were added after lysis to immunoprecipitate non-stimulated TRAIL-receptors. Cell suspensions were incubated for 30 min at 37°C, and the reaction was stopped by the addition of 10 ml ice-cold phosphate buffered saline (6.4 mM Na_2_HPO_4_; 1.5 mM KH_2_PO_4_; 0.14 mM NaCl; 2.7 mM KCl; pH = 7.2). The cells were immediately washed with ice-cold PBS and lysed in 1 ml lysis buffer (30 mM Tris-HCl, pH 7.5, 150 mM NaCl, 1% Nonidet P-40, 10% glycerol, 1 mM phenylmethylsulfonyl fluoride) with complete protease inhibitors (Roche Diagnostics, Almere, the Netherlands) for 15 min on ice. After centrifugation (2,500 × *g*) at 4°C for 10 min, the lysates were pre-cleared with 20 μl Sepharose-6B (Pharmacia, Uppsala, Sweden) for 2 h at 4°C and immunoprecipitated with 30 μl protein-A sepharose beads for 4 h-overnight at 4°C. Beads were washed three times with 1.5 ml lysis buffer, resuspended in standard Western blot sample buffer, and boiled for 5 min. Immunoprecipitated proteins were separated with SDS-PAGE. Western blot analysis for FADD, caspase 8, c-FLIP, DR4, DR5 and TRAIL was performed as described in the SDS-polyacrylamide gel electrophoresis and Western blotting section. Goat HRP-conjugated secondary antibody specific for mouse IgG_1 _and donkey-anti-goat-HRP were used for the detection of caspase 8, FADD, c-FLIP or DR4.

DR4 and DR5-DISC co-immunoprecipitation was performed as described above using the respective Human Genome Sciences antibodies (HGS-ETR1 and TR2J), with some modifications. Briefly, 50.10^6 ^cells per condition were grown in medium harvested and re-suspended in fresh medium. Cells were stimulated with 5 μg/ml antibody in a final volume of 2 ml. The antibodies were added after cell lysis for the control treatment. Cell suspensions were incubated for 15 min at 37°C, and the reaction was stopped by the addition of 10 ml ice-cold phosphate buffered saline (PBS). The cells were immediately washed with ice-cold PBS and lysed in 1 ml lysis buffer (20 mM Tris-HCl, pH 7.5, 150 mM NaCl, 0.2% Nonidet P-40, 10% glycerol, 1 mM phenylmethylsulfonyl fluoride) with complete protease inhibitors (Roche Diagnostics, Almere, the Netherlands) for 30 min on ice. After centrifugation (12,000 × *g*) at 4°C for 10 min, the lysates were pre-cleared with 20 μl Sepharose-6B (Pharmacia, Uppsala, Sweden) for 2 h at 4°C and immunoprecipitated with 50 μl protein-G agarose beads (Roche Diagnostics, Mannheim, Germany) for 3 at 4°C. Beads were washed two times with 1 ml lysis buffer and one time with PBS before resuspension in standard Western blot sample buffer and boiling for 5 min. Immunoprecipitated proteins were separated with SDS-PAGE. Western blot analysis for FADD and c-FLIP was performed as described below. DR4 and DR5 were detected using rabbit anti-DR4 and rabbit anti-DR5 from ProSci Inc. (Poway, CA, USA). Caspase 8 was detected with rabbit anti-caspase 8 (Abcam plc, Cambridge, UK). Goat anti-rabbit HRP-conjugated secondary antibody and rabbit-anti-mouse-HRP were used for the detection of DR4, DR5, FADD, caspase 8 or c-FLIP.

### Apoptosis assay

Apoptosis was assessed using acridine orange (AO) staining, using a method described earlier [36].

### Cytotoxicity assay

The microculture tetrazolium (MTT) assay was used to determine cytotoxicity. SW948 and SW948-TR cells were incubated in a total volume of 200 μl. After an incubation period of 96 h, 20 μl of 5 mg/ml MTT solution diluted in PBS was added for 3.75 h. Subsequently, plates were centrifuged and the supernatant aspirated. After dissolving the formazan crystals by adding dimethyl sulfoxide (Merck, Amsterdam, the Netherlands), plates were read immediately at 520 nm using a microtiter well spectrometer (Bio-Rad microplate reader, Bio-Rad laboratories BV, Veenendaal, the Netherlands). Controls consisted of media without cells. Cell survival was defined as the growth of treated cells compared with untreated cells. IC_50 _was the concentration of drug inhibiting survival by 50%. Mean cytotoxicity was determined in three independent experiments where each condition was performed in quadruplicate.

### Flow cytometry

Analysis of DR4 and DR5 membrane expression was performed as described earlier [36]. For competition experiments with rhTRAIL, SW948 cells (2.10^6^/condition) were harvested, resuspended in 2.5 ml ice cold PBS and incubated on ice with various amounts of rhTRAIL for 1 h. Cells were then washed twice with PBS before fluorescent staining for DR4 and DR5 as described above.

## Results

### rhTRAIL resistance in SW948-TR can be partially explained by its inefficient rhTRAIL-DISC formation

**Figure 1:**
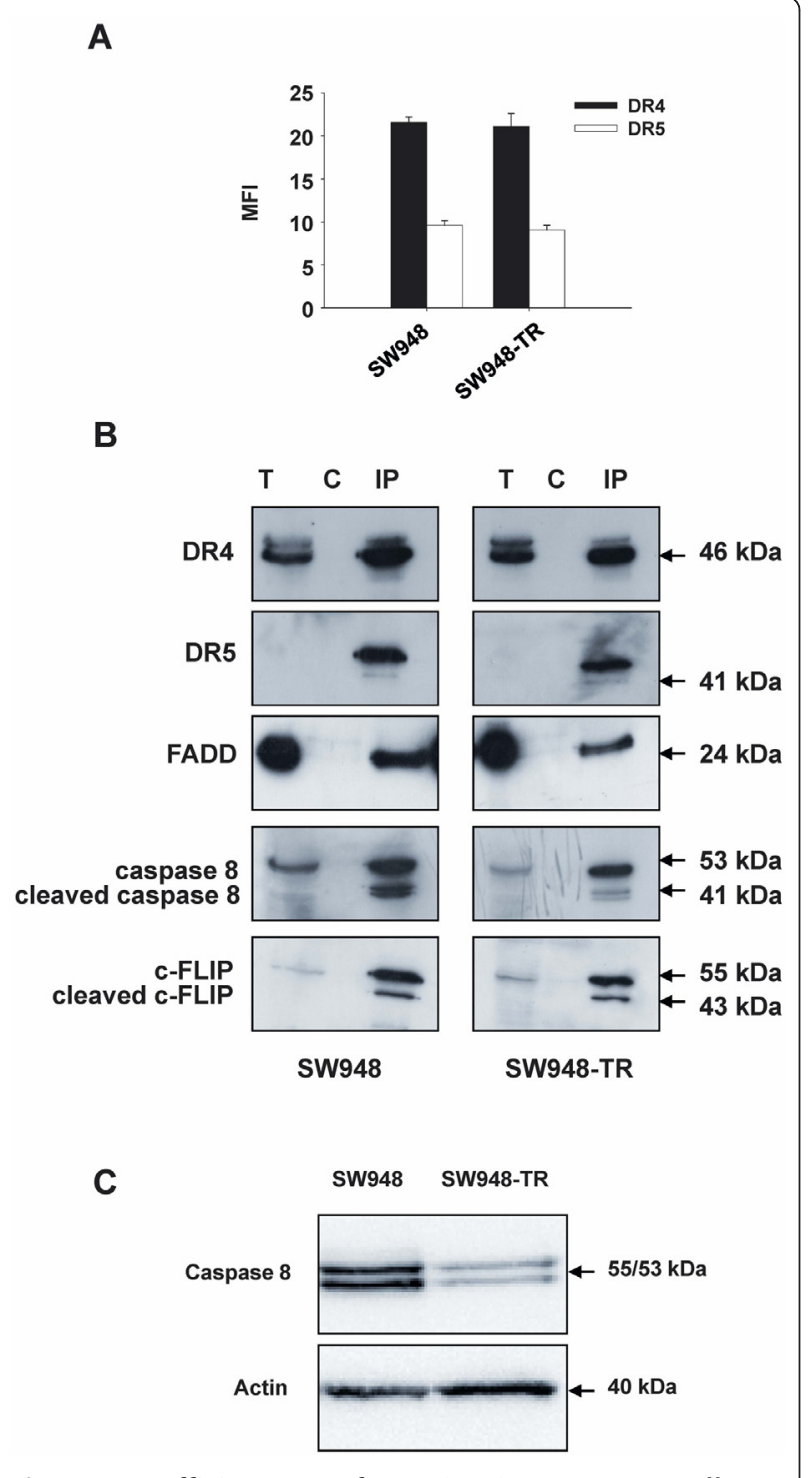
**Inefficient DISC formation in SW948-TR cells**. **A: **Membrane expression of the TRAIL receptors DR4 and DR5 in SW948 and SW948-TR cells as determined by flow cytometry. Values are expressed as the mean fluorescence intensity (MFI) and are mean ± SE of at least three independent experiments. **B: **Analysis of the TRAIL-DISC in SW948 and SW948-TR. Cells were incubated for 30 min with Flag-tagged TRAIL and the assembled DISCs were immunoprecipitated and analyzed by Western blotting using antibodies to DR4, DR5, FADD, caspase 8 and c-FLIP. T = total cell lysates; C = control of immunoprecipitation, IP = immunoprecipitation (After a longer exposure DR5 bands were also detectable in the total cell lysates (T)). **C: **Western blot analysis comparing caspase 8 levels in SW948 and SW948-TR cells. One of at least three independent experiments is shown.

We developed a model for acquired rhTRAIL-resistance consisting of the rhTRAIL-sensitive human colon carcinoma cell line SW948 and its rhTRAIL-resistant sub-line SW948-TR. In these two cell lines, both DR4 and DR5 were expressed at the cell surface; expression of DR4 appeared to be higher (see Figure 1A).

We investigated whether differences in DR functionality could be involved in the SW948-TR cell line's characteristic resistance to rhTRAIL. TRAIL-induced DISC formation was studied. Figure 1B shows that SW948-TR exhibited weaker DISC recruitment of caspase 8 compared with the SW948 cell line. In particular, lower amounts of the cleaved p43/41 form of caspase 8 were measured. Other DISC proteins such as DR4 and DR5, together with FADD and c-FLIP, were equally present in both cell lines. In consistence with our previous findings [33], the SW948-TR cell line was also characterized by lower procaspase 8 expression level (Figure 1C).

### Specific blocking of the individual death receptors indicates that DR4 is more important than DR5 for rhTRAIL-induced apoptosis

**Figure 2:**
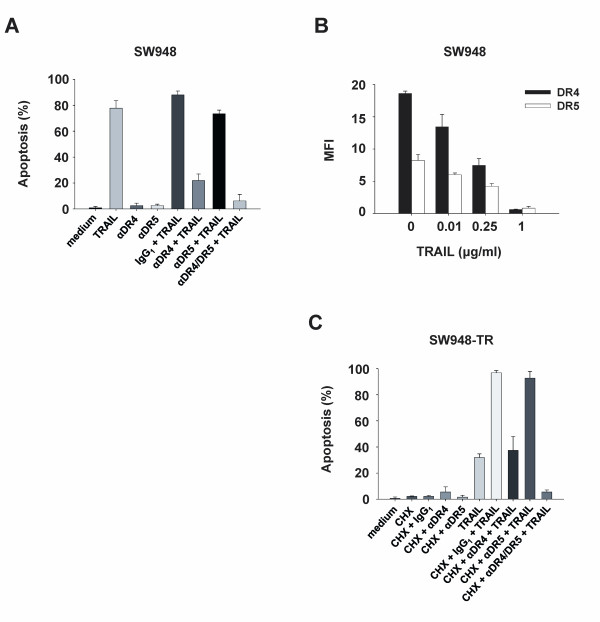
**Important contributions of DR4 to rhTRAIL-induced apoptosis**. **A: **Apoptosis assay in SW948. Cells were pre-incubated with 10 μg/ml antagonistic anti-DR4 (αDR4), anti-DR5 (αDR5) antibodies or IgG1 control for 1 h before 4-5 h rhTRAIL treatment (0.1 μg/ml). Values are mean ± SD of at least three independent experiments. **B: **rhTRAIL binding to DR4 and DR5 in SW948 cells. Cells were incubated with increasing concentrations of rhTRAIL before detection of accessible cell surface DR4 and DR5. Values are expressed as the mean fluorescence intensity (MFI) and are mean ± SE of at least three independent experiments. **C: **Apoptosis assay in SW948-TR. CHX (5 μg/ml) was combined or not with the blocking antibodies 1 h before TRAIL treatment as described in (A).

To evaluate the specific contributions to apoptosis of DR4 or DR5, in the sensitive and resistant cell lines, we used antagonistic TRAIL receptor antibodies to block signaling at the cell surface. In SW948, rhTRAIL-induced apoptosis was reduced by 60% using a DR4 blocking antibody, while blocking DR5 only had a slight effect on rhTRAIL-induced apoptosis (Figure 2A). A competition experiment was performed with SW948 to determine whether rhTRAIL binding to DR4 or DR5 was affected. SW948 cells were pretreated with rhTRAIL and kept on ice to prevent receptor internalization, before detection of DR4 and DR5 using flow cytometry (Figure 2B). A similar decrease in detectable amounts of both death receptors was observed, for a series of increasing concentrations of rhTRAIL. Thus, the binding of rhTRAIL was comparable for each DR in SW948 cells.

Following initial sensitization with the protein synthesis inhibitor CHX, SW948-TR cells were also exposed to rhTRAIL (Figure 2C). Apoptosis was decreased by 60% upon inhibition of rhTRAIL binding to DR4, achieved using a DR4 blocking antibody. Inhibition with a DR5 blocking antibody had almost no effect on rhTRAIL-induced apoptosis. Thus, blocking of DR4 and DR5 with antagonistic antibodies revealed that DR4 was more important than DR5 for rhTRAIL-induced apoptosis; this was true in both SW948 and SW948-TR. In both cell lines, the combination of both antagonistic DR- antibodies inhibited rhTRAIL-induced apoptosis even further than the inhibition achieved using a DR4 blocking antibody alone (15.7% additional reduction for SW948 and 31.9% reduction for SW948-TR, compared with blocking by DR4 antibody). This suggests that although rhTRAIL mostly signals via DR4, both DR4 and DR5 might be important for maximum induction of the apoptotic signal using rhTRAIL.

### rhTRAIL sensitivity of each DR corresponds to sensitivity to agonistic DR4 and DR5 monoclonal antibodies

**Figure 3:**
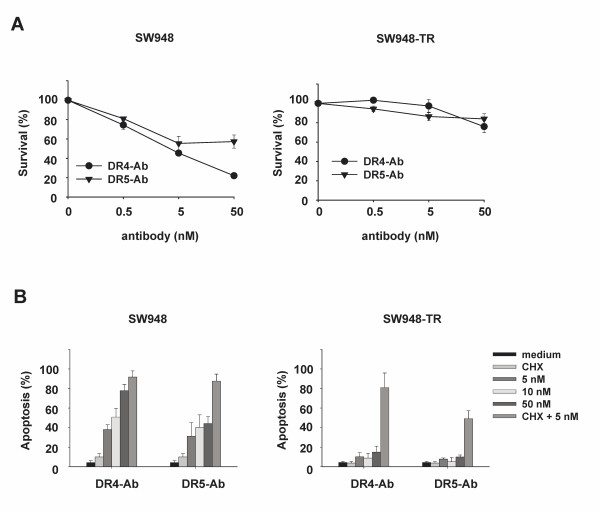
**Sensitivity of SW948 and SW948-TR cells to agonistic DR4 and DR5 antibodies**. **A: **Survival (%) of SW948 and SW948-TR after continuous incubation with agonistic DR4 antibody and agonistic DR5 antibody as measured by cytotoxicity assays. **B: **Apoptosis assay in SW948 and SW948-TR. Cells were pre-incubated with 5 μg/ml CHX for 1 h before incubation with various concentrations of agonistic DR4 antibody (DR4 Ab) and agonistic DR5 antibody (DR5 Ab) for 24 h.

To gain more insight into the functionality of DR4 and DR5 in apoptotic signaling, the effects of agonistic monoclonal DR4 and DR5 antibodies on DR-mediated apoptosis were determined by survival assay. As shown in Figure 3A (left), the rhTRAIL-sensitive cell line SW948 was sensitive to both DR4 and DR5 antibodies. The agonistic DR4 antibody was more effective than the agonistic DR5 antibody, in particular at higher concentrations. SW948-TR cells were resistant to both anti-DR4 and anti-DR5 antibody (Figure 3A, right). These findings were confirmed by apoptosis measured using AO staining (Figure 3B). Thus, the sensitivity of both cell lines to agonistic DR4 or DR5 antibodies seems to partially reflect the individual sensitivity to rhTRAIL of each receptor. Interestingly, the lesser sensitivity to the agonistic DR5 antibody, in comparison with the agonistic DR4 antibody, could be enhanced by CHX in SW948 (Figure 3B, left). CHX could also strongly sensitize SW948-TR cells to agonistic DR4 antibody, and to a lesser extent to agonistic DR5 antibody (Figure 3B, right).

### Upon stimulation with agonistic antibodies, comparable DISC formation occurs at the level of DR4 and DR5 in SW948

**Figure 4:**
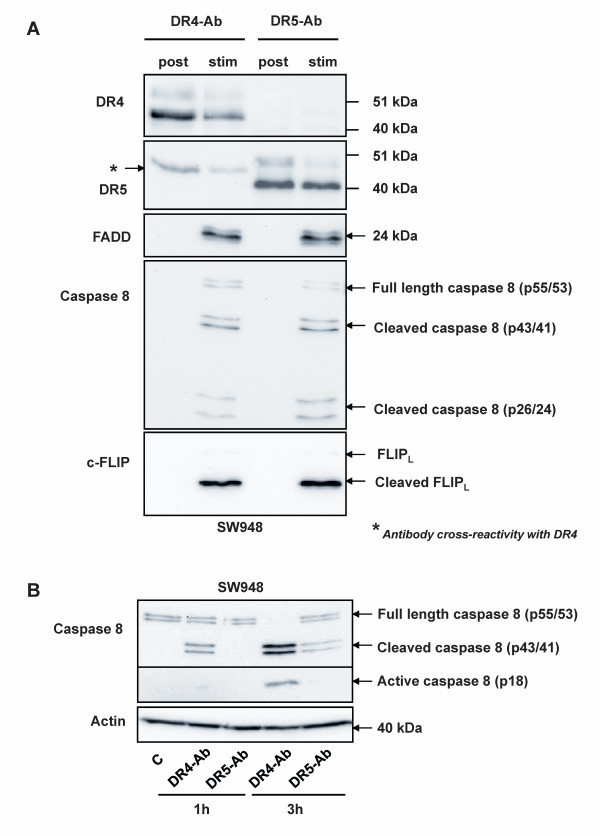
**Equivalent DISC protein recruitment, but not caspase 8 activation, following agonistic DR4 and DR5 antibody treatment**. **A: **Analysis of the DR4 and DR5-DISC in SW948. Cells were incubated for 15 min with agonistic DR4 and DR5 antibodies before co-immunoprecipitation of the associated DISCs using protein G-agarose beads, and subsequent analysis by Western blotting using antibodies directed against DR4, DR5, FADD, caspase 8 and c-FLIP. Post = antibodies added after cell lysis; Stim = cells stimulated with the indicated antibody for 15 min. One representative of at least two independent experiments is shown. **B: **Time-dependent cleavage of pro-caspase 8 in SW948 cells treated with 50 nM agonistic DR4 and DR5 antibodies.

We showed DR4 to be more competent at initiating apoptosis than DR5 when testing SW948 cells. This was demonstrated by stimulating each receptor with rhTRAIL in combination with blocking antibodies, as well as by stimulating each receptor with agonistic antibodies only. To investigate possible differences in DR4-DISC versus DR5-DISC formation, we stimulated SW948 cells with either agonistic DR4 or DR5 antibody, and individually co-immunoprecipitated DR4- and DR5-DISC (Figure 4A). Surprisingly, DISC formation was comparable for both stimuli. Agonistic DR4 antibody was capable of recruiting DR4, FADD, caspase 8 and c-FLIP. Using an antibody directed against the N-terminal fragment of caspase 8, three forms of this protein were detected in the DR4-DISC: the full form, the intermediate p43/41 cleaved form and the p26/24 cleavage product. That the latter form was detectable indicates full cleavage of caspase 8, and thereby release of active caspase 8 from the complex (for comprehensive review see [38]). c-FLIP was mostly present in its intermediate form. The agonistic DR5 antibody recruited DR5 and similar amounts of FADD, c-FLIP and caspase 8 as compared with the agonistic DR4 antibody. Caspase 8 cleavage was also investigated in whole cell lysates of SW948 cells treated with either one of the agonistic DR antibodies (Figure 4B). Within 3 hours, the agonistic DR4 antibody induced full cleavage of the available procaspase 8 into its intermediate and active form. Caspase 8 cleavage in cells stimulated with agonistic DR5 antibody was only partial, as active caspase 8 could not be detected, while unprocessed procaspase 8 molecules were still present after 3 hours.

### Resistance to rhTRAIL, agonistic DR4 and agonistic DR5 antibody can be overcome by the proteasome inhibitor MG-132

**Figure 5:**
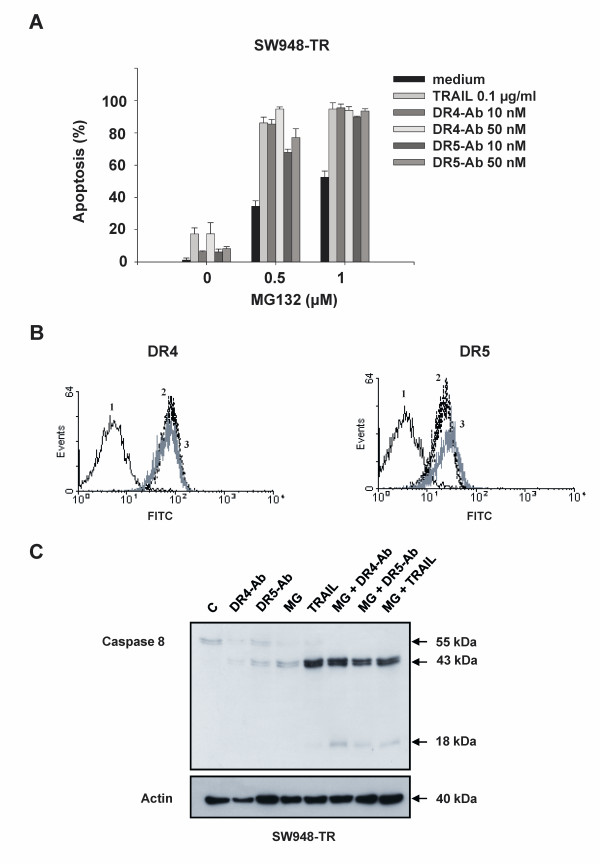
**Sensitization of SW948-TR cells to rhTRAIL, agonistic DR4 and DR5 antibodies by MG-132**. **A: **Apoptosis assay in SW948-TR after 17 h of incubation with MG-132 in combination with rhTRAIL, agonistic DR4 or DR5 antibody. **B: **The effect of MG-132 on DR4 (left figure) and DR5 (right figure) membrane expression as determined by flow cytometry. Receptor expression was detected as the average antigenic density of the whole cell population and resulted in a peak shift to the right. (1 = control; 2 = basal DR4 or DR5 membrane expression level; 3 = DR4 or DR5 expression after exposure to 10 μM MG-132 for 17 h). **C: **Western blot analysis of caspase 8 activation in SW948-TR after 17 h incubation with 1 μM MG-132 in combination with rhTRAIL (0.1 μg/ml), agonistic DR4 or DR5 antibody (50 nM).

Several studies have demonstrated that proteasome inhibition can overcome rhTRAIL-resistance [24,39,40]. Previously, we have observed that SW948-TR could be sensitized to rhTRAIL using the proteasome inhibitor MG-132 [33]. To investigate whether MG-132 sensitized cells in receptor-specific manner, we pre-incubated SW948-TR cells with this compound before treating them with rhTRAIL or agonistic anti-DR4 and -DR5 antibodies. Following MG-132 treatment, we observed that apoptosis induced by either agonistic DR4 or DR5 antibody was enhanced by approximately 50-60%. This effect was similar to the observed enhancement of rhTRAIL-induced apoptosis (Figure 5A). As seen in figure 5B, these effects were not the result of cell membrane DR4 (left figure) or DR5 (right figure) up-regulation by MG-132. Western blot analysis demonstrated that MG-132 increased the intermediate p43/41 cleavage product of caspase 8 (Figure 5C). The combination of MG-132 with rhTRAIL, agonistic DR4 or DR5 antibody led to an almost identical processing of caspase 8 into its active caspase 8 subunit (p18).

### IFN-γ sensitizes SW948-TR to rhTRAIL and agonistic DR4 antibody but not to agonistic DR5 antibody

**Figure 6:**
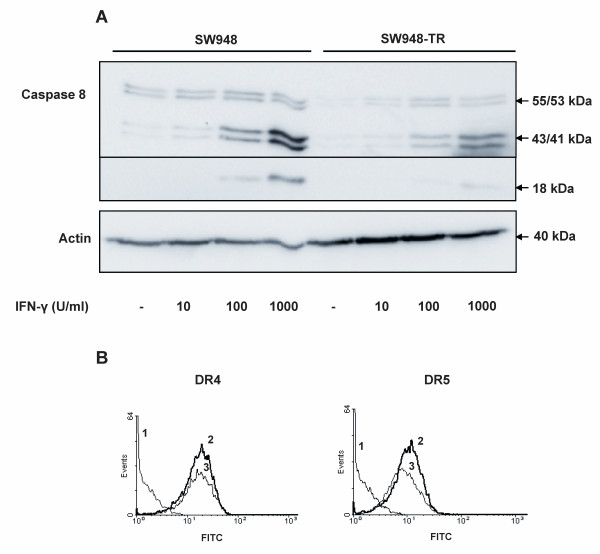
**Increase in caspase 8 levels following treatment of SW948-TR cells with IFN-γ**. **A: **Effects of 48 h incubation with increasing concentrations of IFN-γ on caspase 8 levels in SW948 and SW948-TR. **B: **Effects of IFN-γ on DR4 (left figure) and DR5 (right figure) membrane expression as determined by flow cytometry. Receptor expression was detected as the average antigenic density of the whole cell population and resulted in a peak shift to the right. (1 = control; 2 = basal DR4 or DR5 membrane expression level; 3 = DR4 or DR5 expression after exposure to 1000 units (U)/ml IFN-γ for 48 h).

**Figure 7:**
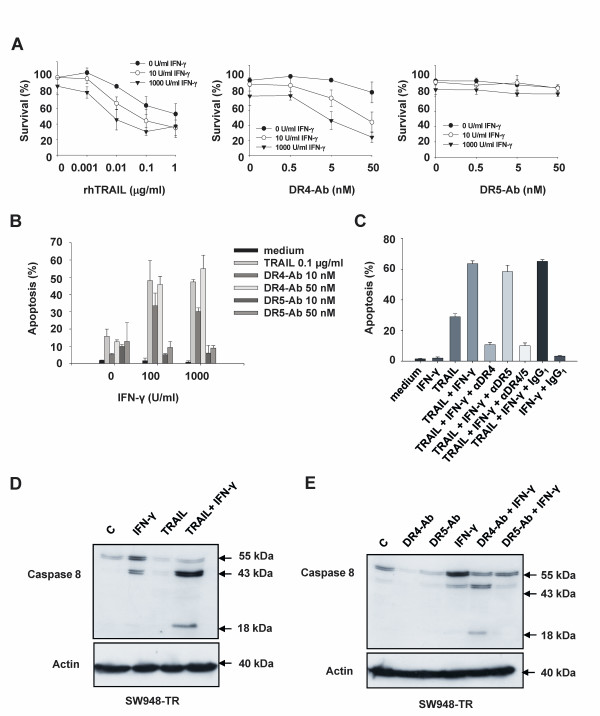
**Stimulation of rhTRAIL- and agonistic DR4 antibody-induced but not agonistic DR5 antibody-induced apoptosis by IFN-γ**. **A: **Survival (%) of SW948-TR cells after continuous incubation with rhTRAIL, agonistic DR4 or DR5 antibody in combination with different concentrations of IFN-γ. **B: **Apoptosis assay of SW948-TR after 48 h incubation with rhTRAIL, agonistic DR4 or DR5 antibody in combination with various concentrations of IFN-γ. **C: **Apoptosis assay in SW948-TR after 48 h incubation with 1000 U/ml IFN-γ. Cells were then incubated with 10 μg/ml antagonistic anti-DR4 (αDR4), anti-DR5 (αDR5) antibodies, both (αDR4/5) or IgG1 control for 1 h before 4-5 h rhTRAIL treatment (0.1 μg/ml). **D: **Western blot analysis of caspase 8 activation in SW948-TR cells. The cells were pre-incubated for 48 hours with 1000 U/ml IFN-γ, then either left untreated or exposed for 4-5 additional hours with rhTRAIL (0.1 μg/ml) before harvest. **E: **Western blot analysis of caspase 8 activation in SW948-TR cells. The cells were pre-incubated for 48 hours with 1000 U/ml IFN-γ, then either left untreated or exposed for 4-5 additional hours with agonistic DR4 antibody (50 nM) or agonistic DR5 antibody (50 nM) before harvest.

Upregulation of caspase 8 by IFN-γ has been described as a mechanism of sensitization to rhTRAIL [41]. Reduced caspase 8 expression levels were detected in SW948-TR as compared with SW948 (figure 1C), which is causative for the observed rhTRAIL resistance in these cells [33]. We therefore hypothesized that IFN-γ might overcome rhTRAIL resistance in SW948-TR. Upon treatment with IFN-γ, caspase 8 protein expression was enhanced in a concentration-dependent manner, in both cell lines (figure 6A). IFN-γ treatment slightly downregulated DR5 but not DR4 surface levels (Figure 6B left and right, respectively). In a previous study we did not observe any additional effect of IFN-γ on rhTRAIL sensitivity in SW948, a cell line that is already extremely sensitive to rhTRAIL [36]. In the current study, survival assays showed that IFN-γ could sensitize SW948-TR to both rhTRAIL and agonistic DR4 antibody but not to DR5 antibody (Figure 7A). Similar results were also seen in an apoptosis assay using AO staining (Figure 7B). Cell survival assays in SW948 indicated that IFN-γ enhanced the sensitivity to agonistic DR4 antibody but not to DR5 antibody (results not shown). TRAIL receptor blocking antibodies were used to determine the relative contribution of each individual DR to IFN-γ induced sensitization to rhTRAIL in SW948-TR. The observed sensitization to rhTRAIL was DR4 specific. The DR4 blocking antibody reduced rhTRAIL-induced apoptosis by 50%, whereas the DR5 blocking antibody only had a minor effect on apoptosis in SW948-TR cells pre-sensitized with IFN-γ (Figure 7C). Western blot analysis of caspase 8 cleavage was performed to gain further insights into the possible mechanism underlying IFN-γ-induced sensitization of DR-mediated apoptosis in SW948-TR cells (Figure 7D and 7E). Treatment with IFN-γ elevated procaspase 8 levels and induced cleavage of procaspase 8 into the intermediate p43/41 product. The combination of IFN-γ with rhTRAIL or agonistic DR4 antibody induced cleavage of caspase 8 to its p18 active form. When cells were treated with IFN-γ and agonistic DR5 antibody, only the intermediate form of caspase 8 (p43/41) could be detected. These results contrasted with the most effective combination; that is, agonistic DR5 antibody with MG-132 (see Figure 5C). This suggests that only DR4-mediated apoptosis benefits from the increase in caspase 8 levels in IFN-γ pretreated cells. We also assessed whether IFN-γ modulated c-FLIP levels. Changes in c-FLIP expression could have affected caspase 8 cleavage in response to the various pro-apoptotic TRAIL receptor ligands (see Additional file 1). Western-blot analysis showed that IFN-γ induced some cleavage of c-FLIP. However, IFN-γ did not change basal c-FLIP levels.

## Discussion

DISC formation is the first step toward apoptosis after engagement of DR4 and DR5 by their ligands. Not surprisingly, resistance to TRAIL-mediated apoptosis is often initiated at the level of the DISC [42]. We observed that upon stimulation with TRAIL, all canonical DISC proteins were recruited to DR4 and DR5 in SW948 and SW948-TR cells. In relation to the other DISC proteins, smaller amounts of cleaved caspase 8 were found in the TRAIL-DISC of SW948-TR cells as compared with SW948 cells. These results were in agreement with the lower basal caspase 8 protein levels found in SW948-TR. Altogether, these findings suggest less active recruitment and processing of caspase 8 in our resistant cell line. We also describe a difference in apoptosis-inducing ability between DR4 and DR5. Despite equivalent initial DISC formation at the level of each receptor, DR4 stimulation achieves superior caspase 8 processing than DR5.

Antagonistic DR4 or DR5 antibodies were used to specifically block the function of their respective receptors during rhTRAIL-induced apoptotic signaling. Experiments with these blocking antibodies proved that DR4 was critical to rhTRAIL-induced apoptotic signaling in both cell lines; DR5 was not. It was previously reported that apoptosis induction in keratinocytes with leucine zipper TRAIL was also mainly mediated by DR4 [43]. In contrast, Kelley *et al*. generated receptor-selective mutants of TRAIL, with three to six ligand amino acid substitutions [17], and found DR5 to be more important for apoptotic signaling than DR4 in cancer cells expressing both receptors (including colon cancer cells). Van der Sloot *et al*. demonstrated that DR5-selective TRAIL variants did not induce apoptosis in cell lines mostly responsive to DR4 stimuli, while they greatly stimulated apoptosis in DR5-responsive cancer cell lines [44]. Little is known about the mechanisms underlying these differences. Blocking of both DR4 and DR5 prevented apoptosis more efficiently than single blocking of DR4, which suggests that DR5 also contributes to rhTRAIL-induced apoptosis. While DR4 was more potent in transducing apoptosis, DR5 might, at least in our model, increase the overall apoptotic stimulus compared with DR4 stimulation alone.

We found that in SW948 and SW948-TR cells, sensitivity to agonistic DR4 and DR5 antibody mostly reflected the sensitivity of each receptor to rhTRAIL. Although the agonistic DR4 antibody was more potent than its anti-DR5 counterpart, agonistic DR5 antibody could induce apoptosis to some extent in SW948 cells, and also in SW948-TR following pre-sensitization with CHX. This strongly suggests that DR5 is functional in both cell lines, as also indicated by rhTRAIL binding and comparable DISC formation between DR4 and DR5. Georgakis *et al*. found that in primary non-Hodgkin's lymphoma samples, an agonistic DR4 antibody was also more effective than an agonistic DR5 antibody [16].

We investigated the ability of each receptor to initiate DISC formation and caspase 8 cleavage in SW948 by co-immunoprecipitating DR4- and DR5-DISC. Similar DISC formation and caspase 8 cleavage were triggered at the level of both receptors, although the rate of caspase 8 processing was much higher in whole cell lysates when cells were stimulated with agonistic DR4 antibody instead of agonistic DR5 antibody. This is consistent with our recent findings that caspase 8 cleavage at the level of DR5-DISC in SW948 is limited due to a lower turn-over of DISC components rather than to decreased DISC formation [45]. Data on such DISC protein turn-over following TRAIL receptor stimulation are limited. However, studies by McDonald *et al*. and Jin *et al*. have suggested that caspase 8 ubiquitination influences processing of the available caspase 8 cellular pool [46,47]. Brief pre-incubation with CHX could also restore DR5-mediated caspase 8 cleavage and sensitivity; a short-lived protein might, therefore, be involved in this process.

IFN-γ is known to increase caspase 8 expression and, consequently, to sensitize cancer cells (including colon cancer cells) to rhTRAIL [24,41,48-50]. Our results are in slight contrast to these findings, as upregulation of caspase 8 by IFN-γ did not further enhance apoptosis induction in the highly sensitive SW948 cells. Caspase 8 levels might not be a limiting factor in these cells, since procaspase 8 recruitment to the DISC is first determined by the amount of FADD molecules available there [51]. IFN-γ did, however, increase apoptosis induction by rhTRAIL and agonistic DR4 antibody in SW948-TR cells, which express lower caspase 8 levels than their parental cells. In SW948-TR, IFN-γ induced a marked increase in both pro- and intermediate forms of caspase 8, which were cleaved to the active form upon DR4 stimulation. Downregulation of caspase 8 levels in SW948 - that is, to a level comparable to that which is normally observed in SW948-TR cells - was sufficient to induce TRAIL resistance, indicating the importance of the caspase-8/c-FLIP ratio in these cells [33]. It is noteworthy that IFN-γ, while inducing an increase in caspase 8 levels, did not change c-FLIP expression in SW948-TR. In contrast, IFN-γ failed to enhance the appearance of active caspase 8 following DR5 stimulation. This was shown using agonistic DR5 antibody, but also rhTRAIL in combination with DR4 blocking antibodies. The slight reduction in DR5 surface expression may explain the lack of increase in DR5-mediated apoptosis.

Unlike IFN-γ, MG-132 markedly increased both DR4- and DR5-mediated sensitivity in SW948-TR cells. Another proteasome inhibitor, bortezomib, was previously reported to enhance the effect of agonistic DR4 and agonistic DR5 antibody in Hodgkin's disease cell lines [18,52]. MG-132 could increase TRAIL-induced apoptosis in both Bax-deficient and proficient colon cancer cells, which is suggestive of a sensitization mechanism independent of the mitochondrial pathway of apoptosis [24]. DR4 and DR5 upregulation by proteasome inhibitors is thought to be a major factor contributing to TRAIL-induced apoptosis sensitization. However, this hypothesis has been subject to an extensive debate [53]. We found that upon MG-132 treatment, DR4-mediated sensitivity increased in the absence of DR4 upregulation. The intermediate form of caspase 8 was upregulated by MG-132, an event which we found was sufficient to restore sensitivity to agonistic DR4 antibody or rhTRAIL following IFN-γ treatment. Importantly, MG-132 also increased caspase 8 cleavage and enhanced DR5-mediated apoptosis. MG-132 has previously been shown to modulate DR5 expression [24,54]. Our flow cytometry analysis only showed negligible DR5 upregulation by MG-132, which again points to a mechanism of sensitization independent of this effect. MG-132 upregulated caspase 8 in SW948-TR, but we demonstrated using IFN-γ that restoring caspase 8 levels was not sufficient to increase DR5-mediated caspase 8 cleavage and apoptosis. Taken together, these results support the existence of additional sensitizing mechanisms of MG-132 at the level of DR5-DISC, beyond DR5 and caspase 8 upregulation. Many of the proteins involved in the DR-mediated apoptotic pathways are regulated by ubiquitination [55], and some of these proteins could play a crucial role in specific regulation of DR5 signaling.

In particular, understanding how MG-132 can overcome DR5-resistance may be of crucial importance, since Johnsen *et al*. previously reported that IFN-γ failed to increase TRAIL-induced apoptosis in 3 out of 8 neuroblastoma cancer cell lines; this result occurred despite caspase 8 upregulation and in the presence of all the proteins known to be necessary for DISC formation [41]. It would be interesting to verify whether DR4 signaling remains functional in the non-responding cells. Furthermore, in view of the large number of agents in clinical development targeting DR5, as opposed to DR4, a deeper knowledge on DR5-mediated signaling regulation is critical for a more rational design of targeted therapies.

## Conclusions

We have demonstrated that cell sensitivity to rhTRAIL signaling via DR4 and DR5 mostly coincides with sensitivity to agonistic DR4 and DR5 antibodies, respectively. In SW948-TR cells, apoptosis induced by DR4 stimuli seems to be limited by the lesser amounts of caspase 8 available (in comparison with the parental cell line). Resistance to DR5-mediated apoptosis in SW948-TR cells most likely stems from a combination of at least two mechanisms: (1) low caspase 8 levels and (2) sub-optimal capacity to process the existing caspase 8 at the level of this receptor, which was also seen in the parent cell line. Only MG-132 was able to restore generation of active caspase 8, upon DR5-stimulation, in the SW948-TR line. These results underscore the DR-specificity of drug combinations and demonstrate the presence of different resistance mechanisms at the level of DR4 and DR5.

## Competing interests

The authors declare that they have no competing interests.

## Authors' contributions

CvG and BP performed most of the experiments and drafted the manuscript. PL carried out the MTT assays. EdV and SdJ participated in the design and coordination of the study and helped to draft the manuscript. All authors read and approved the final manuscript.

## Pre-publication history

The pre-publication history for this paper can be accessed here:

http://www.biomedcentral.com/1471-2407/11/39/prepub

## Supplementary Material

Additional file 1 **Effects of IFN-γ on c-FLIP cleavage**. Western blot analysis of c-FLIP levels in SW948-TR after 48 h preincubation with 1000 U/ml IFN-γ in combination with rhTRAIL (0.1 μg/ml), agonistic DR4 or DR5 antibody (50 nM) treatment for 5 h. Click here for file
