# Supplementary figures and images for: Modulation of TRAIL resistance in colon carcinoma cells: Different contributions of DR4 and DR5
Source: BMC Cancer. 2011 Jan 27;11:39. doi: 10.1186/1471-2407-11-39 (PMC3045356; doi:10.1186/1471-2407-11-39)

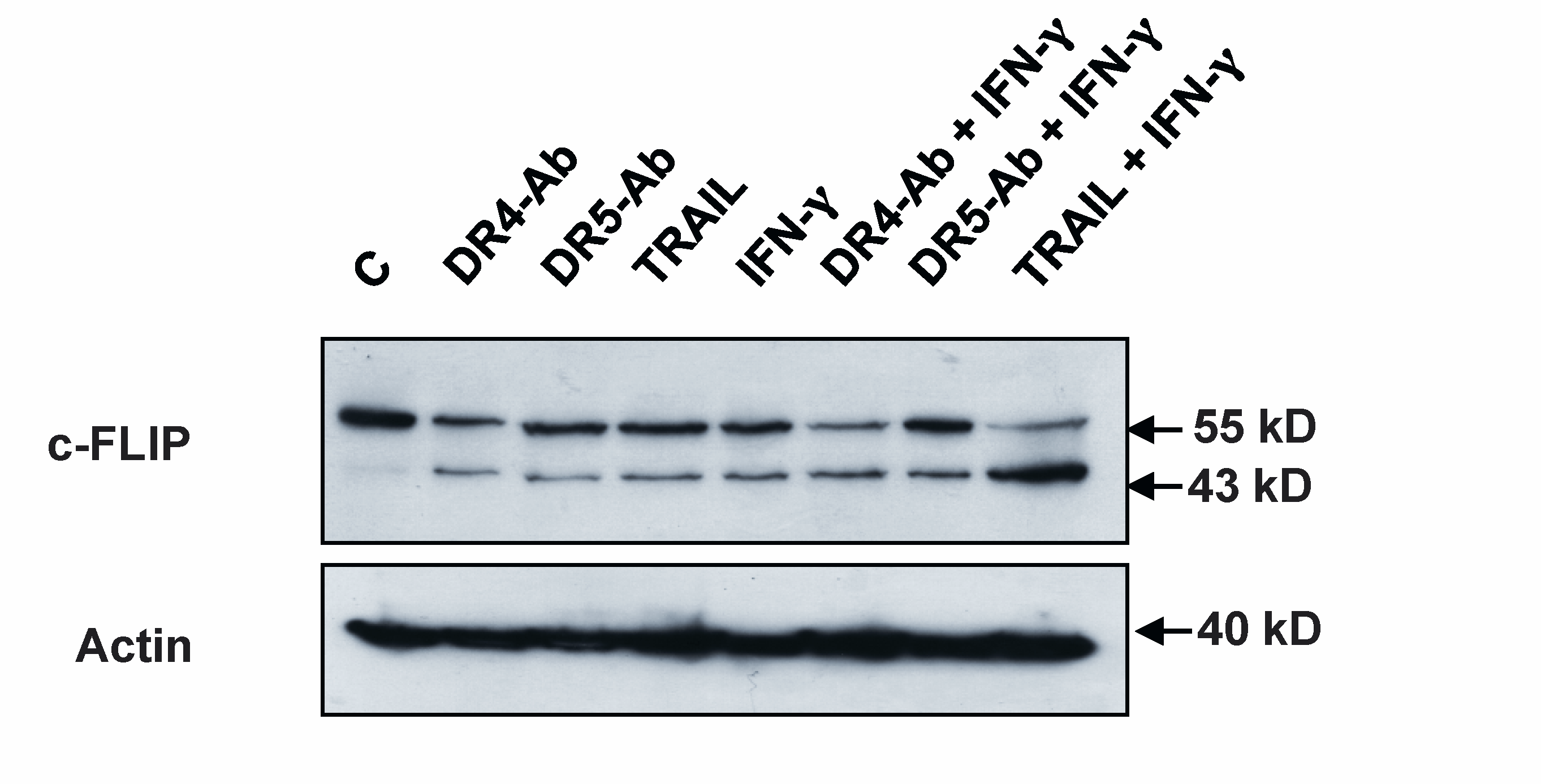

Supplement: Additional file 1 — Effects of IFN-γ on c-FLIP cleavage. Western blot analysis of c-FLIP levels in SW948-TR after 48 h preincubation with 1000 U/ml IFN-γ in combination with rhTRAIL (0.1 μg/ml), agonistic DR4 or DR5 antibody (50 nM) treatment for 5 h. [file 1471-2407-11-39-S1.TIFF]
